# The correlation between the cerebroplacental ratio and fetal arterial blood gas in appropriate-for-gestational-age fetuses: A cross-sectional study

**DOI:** 10.18502/ijrm.v19i9.9714

**Published:** 2021-10-10

**Authors:** Ashraf Jamal, Vajiheh Marsoosi, Fatemeh Sarvestani, Neda Hashemi

**Affiliations:** ^1^Department of Perinatology, Tehran University of Medical Sciences, Tehran, Iran.; ^2^Department of Perinatology, Endometriosis Research Center, Rasoul Akram Hospital, Iran University of Medical Sciences, Tehran, Iran.

**Keywords:** Umbilical cord blood, Color Doppler ultrasonography, Gestational age.

## Abstract

**Background:**

The cerebroplacental ratio (CPR) is an important index for predicting adverse pregnancy outcomes in small-for-gestational-age and appropriate-for-gestational-age fetuses.

**Objective:**

To find out whether there is an association between the CPR level and the blood cord gases analysis in appropriate for gestational age fetuses.

**Materials and Methods:**

This cross-sectional study included 347 pregnant women at the gestational age of 37-40 wk. Patients had an appropriate-for-gestational-age fetus confirmed from their first ultrasonography results. Participants were divided into two groups based on their CPR, measured before delivery. Finally, after delivery, arterial blood gas level and the incidence of emergency cesarean section, intrapartum fetal distress and neonatal intensive care unit admissions were compared between the two groups.

**Results:**

Fifty-four (15.6%) cases had a CPR below the detection limit of the assay. The incidence of fetal distress, emergency cesarean section, neonatal hospitalization in the neonatal intensive care unit, and pH < 7.2 were significantly lower in women with CPR ≥ 0.67 multiples than in women with a CPR < 0.67 multiples of the median.

**Conclusion:**

The third-trimester CPR is an independent predictor of stillbirth and perinatal mortality and morbidity. The role of UA/MCA Doppler and the CPR in assessing the risk of adverse pregnancy outcomes should be evaluated prospectively.

## 1. Introduction

The cerebroplacental ratio (CPR) is an important index for predicting adverse pregnancy outcomes in small-for-gestational-age (SGA) and appropriate-for-gestational-age (AGA) fetuses. The CPR is calculated by dividing the pulsatility index (PI) of the middle cerebral artery (MCA) by that of the umbilical artery (UA) (1).

The CPR, which was first introduced in 1987, is recognized as a more sensitive Doppler index to predict perinatal outcomes, as it shows the status of both the placenta and fetus. Doppler indices for CPR measurements include the resistance index, the PI of the UA, and Doppler waveform indices of MCA. However, the CPR is not routinely measured in clinical practice (2).

Intrapartum hypoxia is the cause of nearly 10-15% of cases of cerebral palsy. Despite the clinical importance of this condition, antenatal identification of fetuses at risk of cerebral palsy and other hypoxia-related outcomes remains a challenge. Hence, a prediction of fetal blood gas imbalance could be helpful for identifying the associated risks and preventing their consequences. Fetal CPR, measured within 72 hr after delivery, can help identify fetuses that are likely to require obstetric intervention for intrapartum fetal compromise (3).

In this study, we aimed to determine the correlation between the CPR and neonatal outcomes in AGA fetuses.

## 2. Materials and Methods

This cross-sectional study was conducted from December 2016 to February 2018 on young pregnant women referred to Dr. Shariati Hospital, a referral hospital affiliated to Tehran University of Medical Science, Tehran, Iran.

A total of 403 pregnant women were recruited who were at the gestational age (GA) of 37-40 wk and had an AGA fetus according to their first ultrasonography. The exclusion criteria were a history of multiple pregnancies, history of preterm birth, fetal growth restriction, and fetal abnormalities. However, 56 women were later excluded due to a lack of sampling and other limiting conditions.

Finally, participants were divided into two groups according to their CPR level, as measured before delivery. After delivery, the arterial blood gas (ABG) level, and the incidence of emergency cesarean section, intrapartum fetal distress and neonatal intensive care unit admissions were compared between the two groups.

Color Doppler sonography of the UA and MCA was performed by a perinatologist before delivery via ultrasonography device (Phillips, Affinity 50, USA). The CPR was calculated by dividing the PI of the MCA by that of the UA.

The ABG was measured in the hospital's laboratory and the total number of cases who were admitted to the neonatal intensive care unit (NICU) was recorded. Fetal distress was evaluated with FHR monitoring.

### Ethical considerations

This study was approved by the ethics committee of Tehran University of Medical Sciences (Code: IR.TUMS.MEDICINE.REC.1396.4301). All patients provided informed consent prior to the study.

### Statistical analysis

Data analysis was performed using Social Sciences statistical package v. 19 (SPSS, Inc, Chicago, IL). Descriptive analyses were performed including means, and standard deviations. The baseline characteristics of the two groups were compared using the student's *t* test. Differences in the pregnancy outcomes of the two groups were analyzed using the Chi-square test. P < 0.05 were considered significant.

## 3. Results

In this study, 403 pregnant women who met the inclusion criteria of the study were enrolled. During the study, 56 of these women were excluded (due to inability to receive an ABG measurement from the fetus, sample loss or im-proper sampling).

Patients were divided into two groups based on their CPR level (cut-off: 0.67 MOM).

Participants in the two groups did not differ significantly in terms of demographic characteristics including maternal age, GA at the time of ultrasonography, and GA at the time of delivery (Table I).

The incidences of fetal distress, emergency cesarean section, neonatal hospitalization in the NICU, and pH < 7.2 were significantly lower in than in women with a CPR-MOM < 0.67 (Table II). Table III shows the lowest and highest UA and MCA PI, CPR, CPR_ MOM and pH of UA.

**Table 1:** Demographic characteristics of the participants in the two study groups


	**CPR-MOM ≥ 0.67 (n = 293)**	**CPR-MOM < 0.67 (n = 54)**	**p-value**
**Maternal age (yr)**	30.2 ± 5.6	28.8 ± 6.5	0.148
**GA at the time of ultrasonography (wk)**	38.3 ± 1.1	38.1 ± 1.0	0.414
**GA at the time of delivery (wk)**	38.5 ± 1.1	38.3 ± 1.1	0.498
Data presented as Mean ± SD. Student's *t *test, CPR-MOM: Cerebroplacental ratio-multiples of the median, GA: Gestational age, CS: Cesarean section, NICU: Neonatal intensive care unit			

**Table 2:** Comparison of the pregnancy outcomes between two study groups


**Variables**	**CPR-MOM ≥ 0.67 (n = 293)**	**CPR-MOM < 0.67 (n = 54)**	**p-value**
**Fetal distress**	17 (5.8)	30 (55.6)	< 0.001*
**Emergency CS**	16 (5.5)	28 (51.9)	< 0.001*
**Neonatal hospitalization in NICU**	1 (0.3)	6 (11.1)	< 0.001*
**Cord blood PH**	1 (0.3)	39 (72.2)	< 0.001*
**Umbilical artery pH**	7.31 ± 0.05	7.18 ± 0.06	< 0.001**
**Birth weight (g)**	3195.1 ± 364.7	3122.0 ± 376.0	0.179**
**Birth weight centile**			
**10-25${}^{\mathrm{th}}$**	25 (8.5)	9 (16.7)	
**26-50${}^{\mathrm{th}}$**	91 (31.1)	11 (20.4)	
**51-75${}^{\mathrm{th}}$**	112 (38.2)	24 (44.4)	
**76-90${}^{\mathrm{th}}$**	65 (22.2)	10 (18.5)	0.134*
*Data presented as n (%) (Chi-squared test). **Data presented as Mean ± SD. (Student’s *t* test). CS: Cesarean section, NICU: Neonatal intensive care unit, CPR-MOM: Cerebroplacental ratio multiples of the median, pH: Potential of hydrogen			

**Table 3:** Maximum and minimum of UA and MCA PI, CPR and UA PH


	**MCA-PI**	**UA-PI**	**CPR**	**CPR MOM**	**PH of UA**
**Mean ± SD**	1.56 ± 0.36	0.91 ± 0.15	1.76 ± 0.53	0.98 ± 0.3	7.29 ± 0.07
**Minimum **	0.85	0.52	0.86	0.47	6.96
**Maximum **	2.48	1.69	3.77	2.23	7.48
PI: Pulsatility index, UA: Umbilical artery, MCA: Middle cerebral artery, CPR: Cerebroplacental ratio, CPR MOM: Cerebroplacental ratio multiples of the median, pH: Potential of hydrogen					

## 4. Discussion 

The results of all variables were different between the two groups. Based on the findings, it can be concluded that a low CPR is associated with a low Apgar score, low pH in the ABG of the neonate, and an increased rate of emergency cesarean section and NICU admission. As a result the third-trimester CPR is an independent predictor of stillbirth and perinatal mortality and morbidity.

Numerous studies have shown that a low CPR indicates redistribution of fetal blood flow (brain-sparing) and predicts adverse outcomes in neonates. The abnormal fetal growth velocity, NICU admission, emergency cesarean section for fetal distress, intraventricular hemorrhage, hypoxic ischemic encephalopathy, necrotizing enterocolitis, bronchopulmonary dysplasia, sepsis, and even death are some of the adverse fetal outcomes (4-6).

Term infants weighing > 10${}^{\mathrm{th}}$ percentile of GA are considered AGA. Although AGA fetuses are generally healthy, some of them may suffer from placental insufficiency and fail to reach their ideal genetic weight (7-9). AGA fetuses or those with a late onset growth retardation (> 34 wk of gestation) and an abnormal CPR are at risk of fetal distress in the active phase of labor and may require emergency cesarean section. In these cases, the cord blood pH decreases, and the incidence of NICU admission increases compared with normal-CPR fetuses (1).

The CPR is a preliminary diagnostic measure of pregnancy complications, which can be compared to the biophysical profile score and UA/MCA Doppler studies. The CPR can be used as an independent tool in evaluating of third-trimester fetuses, regardless of UA and MCA findings (1).

Detection of stillbirth in prolonged pregnancies is linked to late placental insufficiency and fetal hypoxemia. Therefore, fetal Doppler monitoring may improve the management of fetal condition, although the literature suggests conflicting results. The CPR can help to detect fetal hypoxemia through two mechanisms of reduced resistance in MCA (brain-sparing effect) and increased placental resistance. The CPR has been shown to be more important than Doppler indices of MCA and UA alone in predicting adverse fetal outcomes, associated with growth restriction and prolonged pregnancy (10-12).

There is much literature in this field, discussing the pros and cons of the CPR calculation and its features. A number of studies have shown an association between the CPR and growth rate as well as birth weight (4, 13, 14).

Moreover, some researchers showed that the CPR is a major independent predictor of stillbirth and perinatal mortality, and the CPR has the highest sensitivity in the prediction of both intrapartum abnormal fetal heart rate and adverse neonatal outcomes in prolonged pregnancies (5, 6, 15, 16).

However, some believe that there is no relationship between pregnancy outcomes and CPR in prolonged pregnancies (10).

The literature suggests that fetuses with CPRs < 0.67 are at an increased risk of intrapartum compromise and are less likely to be delivered vaginally (17). These finding are in line with the results of our study.

They revealed that the CPR is a marker of impaired fetal growth velocity and adverse pregnancy outcomes, even in fetuses whose size is considered appropriate based on conventional biometry (12, 18).

The findings of this study demonstrate that a low CPR in AGA fetuses is an important marker of low neonatal pH.

## 5. Conclusion 

The CPR is an important factor in predicting the morbidity and mortality of infants. And a low CPR, even in normal weight fetuses, can be a sign of hypoxemia and placental insufficiency. However, this claim needs further investigation.

##  Conflict of Interest

The authors declare that there is no conflict of interest.
